# Correlation of muscle strength, information processing speed and cognitive function in the elderly with cognitive impairment——evidence from EEG

**DOI:** 10.3389/fnagi.2025.1496725

**Published:** 2025-01-20

**Authors:** Xin Xin, Qing Liu, Shuqi Jia, Shufan Li, Peng Wang, Xingze Wang, Xing Wang

**Affiliations:** ^1^School of Physical Education, Shanghai University of Sport, Shanghai, China; ^2^School of Physical Education, Huzhou University, Huzhou, China

**Keywords:** muscle strength, information processing speed, cognitive impairment, the elderly, EEG

## Abstract

**Objective:**

This study investigates the interplay between muscle strength, information processing speed, EEG-specific biomarkers, and cognitive function in elderly individuals with cognitive impairments, emphasizing the mediating roles of information processing speed and EEG-specific biomarkers.

**Method:**

A cross-sectional study design was employed to recruit 151 elderly participants. The participants underwent grip strength and 30-s sit-to-stand tests to assess muscle strength, completed the Trail Making Test part A (TMT-A) and the Symbol Digit Modality Test (SDMT) to evaluate information processing speed, and utilized the Montreal Cognitive Assessment (MOCA) to gauge cognitive function. Additionally, EEG signals were recorded for 5 min to capture neural activity.

**Results:**

The difference in information processing speed among elderly individuals with varying degrees of cognitive impairment was statistically significant (*p* < 0.001). A significant negative correlation was observed between the MoCA score and the time consumption of TMT-A (*r* = −0.402, *p* < 0.01), and a significant positive correlation was found between the MoCA score and the SDMT score (*r* = 0.609, *p* < 0.01). Grip strength was negatively correlated with the time consumption of TMT-A (*r* = −0.336, *p* < 0.01) and positively correlated with the SDMT score (*r* = 0.336, *p* < 0.01). A significant negative correlation was found between the 30-s sit-to-stand test and the time consumption of TMT-A (*r* = −0.273, *p* < 0.01), and a significant positive correlation was observed between the 30-s sit-to-stand test and the SDMT score (*r* = 0.372, *p* < 0.01). Additionally, we observed that the α1 power value indicators were significantly correlated with the MoCA score, the time consumption of TMT-A, and the SDMT score (all *p* < 0.01). The α1 power values at F7 + F8 and T5 + T6 were identified as sensitive EEG indicators for muscle strength and information processing speed. The EEG-specific indicators (*B* = 0.019, 95% CI: 0.003, 0.047) and information processing speed (*B* = 0.137, 95% CI: 0.096, 0.292) were found to partially mediate the relationship between grip strength and MoCA scores, with information processing speed exerting a stronger mediating effect.

**Conclusion:**

Specific patterns were observed in the EEG of elderly individuals with cognitive impairments, which could objectively assess the risk of cognitive decline in this population. Muscle strength, information processing speed, and EEG-specific biomarkers were closely associated with cognitive function in elderly individuals. The potential pathway of interaction—muscle strength → EEG-specific biomarkers → information processing speed → cognitive function—provides valuable insights into advancing the field of cognitive research in the elderly.

## 1 Introduction

Cognitive impairment is a common problem in the elderly, often characterized by varying degrees of decline in attention, memory, executive function, language and visuospatial ability. Mild cognitive impairment (MCI) is the intermediate stage between health and dementia (Anderson, 2019), and the prevalence of MCI among people in communities worldwide exceeds 15% (Petersen et al., 2018).

Over time, the severity of cognitive impairment can progress from mild cognitive impairment (MCI) to Alzheimer’s disease or dementia, with the annual conversion rate of MCI to dementia averaging approximately 10%, significantly higher than the general population’s annual incidence rate of 1–2% (Petersen et al., 2018; Anderson, 2019). MCI, a clinical condition characterized by cognitive impairment, leads to deficits in social and cognitive abilities, impeding task performance and reducing both the independence and autonomy of elderly individuals (Kelley and Petersen, 2007). Moreover, the reliance on care and the progressive decline in cognitive function represent long-term challenges for both patients and caregivers, potentially imposing a substantial burden on families and society (Connors et al., 2019). Research has demonstrated that the ability of elderly individuals to process new information and make swift decisions declines progressively with age (Murman, 2015). Information processing speed (IPS) is defined as “the amount of time required to process a given set of information or the volume of information that can be processed within a specified time interval” (Chiaravalloti et al., 2013), and it plays a crucial role in cognitive functions such as memory, attention, and executive function (Poggel and Strasburger, 2004). Slow IPS is a hallmark feature of cognitive impairment (Poggel and Strasburger, 2004) and is frequently identified as a primary cause of attention deficits, particularly evident in individuals with traumatic brain injury (TBI), dementia, Parkinson’s disease, and multiple sclerosis (Lev et al., 2014). Most scholars believe that preventing and delaying cognitive decline in the elderly should focus on enhancing the core element of information processing speed. Based on these findings, the study proposes Hypothesis H1: Information processing speed directly influences the cognitive function of elderly individuals with cognitive impairments.

Electroencephalography (EEG), a technique that directly measures neurological activity with high temporal resolution, has emerged as a promising tool for detecting neurobiomarkers associated with mild cognitive impairment (MCI) and Alzheimer’s disease (AD) (Meghdadi et al., 2021; Vecchio et al., 2020; Vecchio et al., 2018). A growing body of evidence supports the use of EEG in distinguishing patients with MCI and AD from healthy individuals (Farina et al., 2020; Cassani et al., 2017; Hatz et al., 2015; Musaeus et al., 2018). Previous studies involving EEG recordings from patients with MCI and AD have demonstrated that their neurological changes are relatively consistent compared to healthy individuals, including a reduction in *α*-and *β*-band activity and an increase in *δ*-and *θ*-wave oscillations. These changes are strongly correlated with cognitive function, making them potential neurobiomarkers for the early detection of AD (Farina et al., 2020; Monllor et al., 2021). Additionally, the amplitudes of α-band activity have been shown to reflect the orientation and tactile processing of visuospatial attention (Foster et al., 2017; Haegens et al., 2012). Based on these findings, the study proposes Hypothesis H2: A direct relationship exists between specific EEG indicators, information processing speed, and cognitive function in elderly individuals with cognitive impairments.

The study identified that deficits in physical activity behaviors are prevalent in individuals with MCI and AD (Albers et al., 2015), including reduced grip strength, slower gait, and impaired hand flexibility (De Paula et al., 2016; Kuo et al., 2023). There is a growing consensus that exercise can effectively prevent and delay cognitive decline in the elderly (Burtscher and Burtscher 2024). Previous findings from our research team also established correlations between muscle strength, working memory, and cognitive function in the elderly (Li et al., 2023; Cai et al., 2023). Muscle strength has been identified as a predictor of information processing speed (Russ et al., 2022), which is a key dimension of cognitive function. Information processing in the brain is governed by oscillatory activity. A wealth of empirical studies has emphasized the critical role of synchronous EEG activity in various aspects of cognitive function, including perception, attention, learning, memory, motor control, and higher-level goal-directed behavior (Cheron et al., 2016; Niv, 2021). Neurophysiological studies investigating cognitive function have revealed considerable uncertainty regarding the functional significance of activities in different frequency bands and their interrelationships (Eronen and Bringmann, 2021). Therefore, there is an urgent need for a systematic interpretation of the oscillations and brain activity associated with different frequency bands involved in cognitive and motor control. Based on these findings, the study proposes Hypothesis H3: Muscle strength has a direct influence on specific EEG indicators, information processing speed, and cognitive function in elderly individuals with cognitive impairments.

A review of existing literature reveals that the neurobiological characteristics of MCI include reduced perfusion in the temporal and parietal cortices, medial temporal lobe atrophy, a reduction in Aβ42 levels, and elevated levels of hyperphosphorylated tau in the cerebrospinal fluid (Susianti et al., 2024; Huang et al., 2018; Cai et al., 2023; Gonzalez-Ortiz et al., 2024). MCI is frequently characterized by mild cognitive impairment, representing an optimal window for the implementation of both pharmacological and non-pharmacological interventions aimed at delaying the progression of Alzheimer’s disease (AD) and dementia. Therefore, there is an urgent need for effective identification and intervention in MCI among the elderly.

Cognitive decline in the elderly is attributed to multiple factors, and there is likely a strong interrelationship between muscle strength, information processing speed, brain function, and cognitive decline. Elucidating the nature of this relationship could offer valuable insights into strategies for delaying cognitive decline in the elderly. In light of this, it is crucial to explore the interrelationships among these factors and examine potential shared pathways. To test the three hypotheses proposed in this study (H1, H2, H3) and further provide complementary evidence for the proposed pathways, this study will adopt a cross-sectional design and apply structural equation modeling to examine the relationships among muscle strength, information processing speed, brain function, and cognitive decline. Additionally, it will explore the mediating roles of information processing speed and brain function in the relationship between muscle strength and cognitive function, providing a foundation for the early identification and prevention of age-related cognitive decline in the elderly.

In light of this, the present study aimed to answer the following questions: Is there a pairwise correlation between muscle strength (grip strength, 30-s sit-to-stand), information processing speed (TMT-A, SDMT), and EEG? What EEG indicators mediate the relationship between muscle strength (grip strength, 30-s sit-to-stand) and information processing speed, and what is the underlying pathway? This study is part of a decade-long brain science research program led by Professor Wang Xing’s team, which aims to elucidate the potential cognitive and neural mechanisms by which exercise may enhance cognition, thus providing a foundation for future causal research and offering potential solutions for the clinical treatment of elderly patients with cognitive impairments.

## 2 Research objects and methods

### 2.1 Recruitment of participants

**Figure 1:**
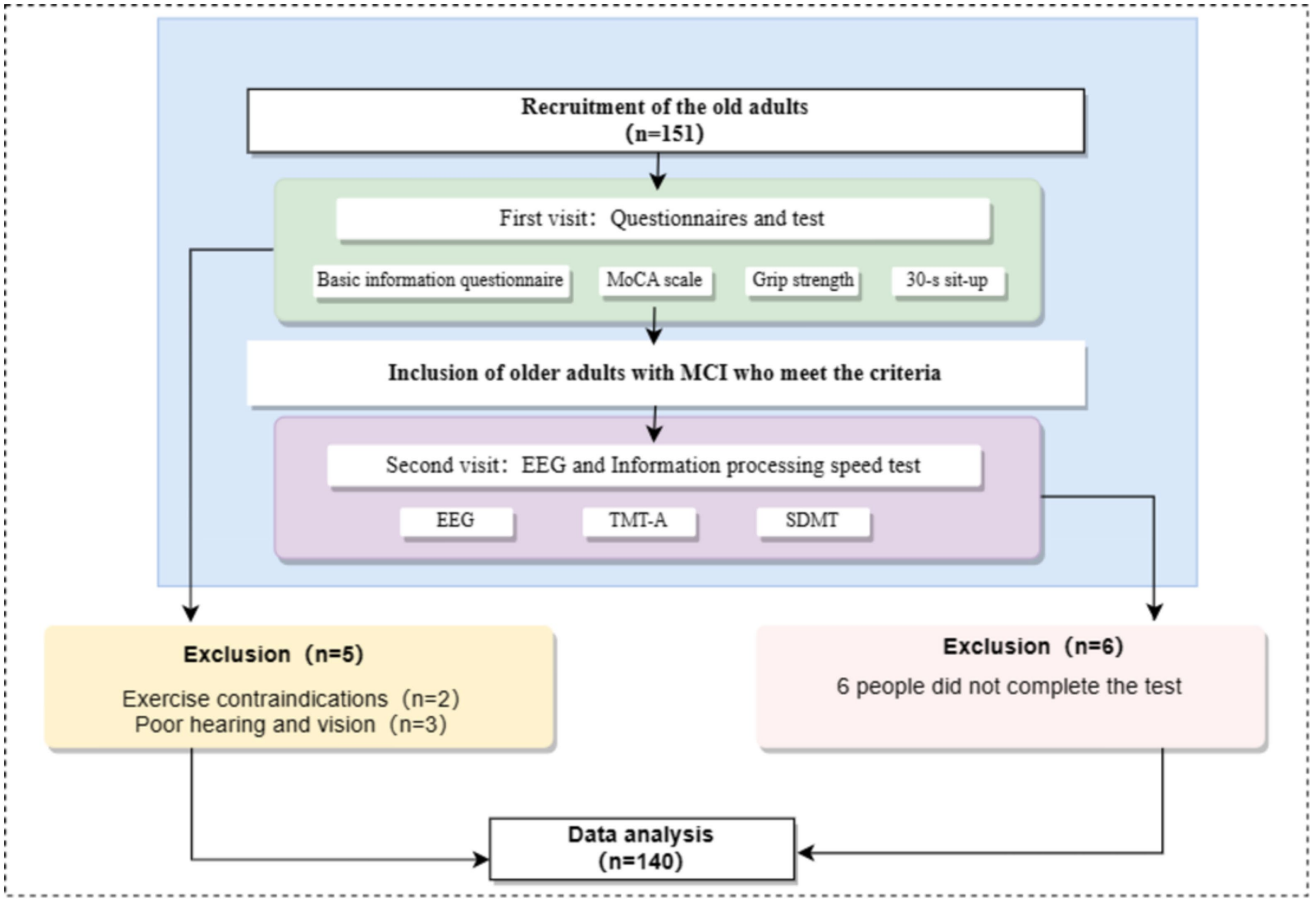
Flowchart of participants recruitment.

Through convenience sampling, 151 elderly participants were recruited in some communities in Songjiang District and Jinshan District of Shanghai. This study met the ethical requirements of the latest version of the Declaration of Helsinki and was approved by the Ethics Committee of Shanghai Sport University (102772020RT060). Participants were recruited by trained researchers who put up the recruitment posters and distributed recruitment leaflets. The participants recruitment process was shown in Figure 1.

Inclusion criteria: (1) Participants aged 60 years and above; (2) Participants with right handedness; (3) Participants in good physical condition; (4) Participants without serious cardiovascular disease; (5) Participants with normal vision and hearing; (6) Participants with normal mental state, who were able to communicate verbally, and willing to cooperate with the completion of the survey; (7) Participants willing to sign the informed consent。.

Exclusion criteria: (1) Participants with severe cardiovascular disease or major organic disease; (2) Participants with severe muscle disease who were unable to stand for long periods of time; (3) Participants with exercise contraindications; (4) Participants with poor vision or hearing who are unable to complete the examination; (5) Participants with long-term or recent use of psychotropic drugs, the drugs that affected physical activity, cholinergic inhibitors, and other related drugs.

### 2.2 Test process

**Figure 2:**
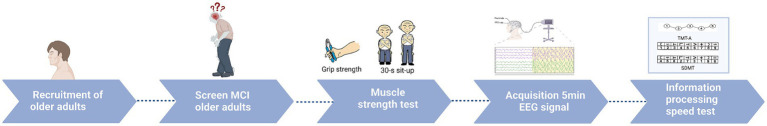
Flowchart of test process.

All the tests took place between 13:30 and 16:30. All participants were required to visit the laboratory twice. When the participants entered the laboratory for the first time, they should be explained about the experimental procedure and they needed to fill out the basic information table and the “Montreal Cognitive Assessment” (MoCA). In addition, the participants should complete the height, weight, grip strength tests and the 30-s sit-to-stand test. When the participants entered the laboratory for the second time, the EEG signal collection and information processing speed test should be completed. The test process was shown in Figure 1. Participants should avoid doing strenuous exercise and drinking caffeinated or alcoholic beverages 24 h before the test. All participants voluntarily participated in the experiment, signed informed consent, and the study followed the ethical requirements of the latest version of the Declaration of Helsinki (Figure 2).

#### 2.2.1 Test tools

##### 2.2.1.1 Self-compiled basic information questionnaire

The basic information of the participants was collected, including name, age, gender, BMI, marital status, education level, occupation, place of residence, social activities, medical history, drug use, whether they had exercise contraindications, smoking or drinking history, and whether there existed the decline in their hearing and vision.

##### 2.2.1.2 MoCA scale

The scale had been widely used to assess cognitive function in the elderly in eight areas: visuospatial/executive function, naming, memory, attention, language fluency, abstract thinking, delayed recall, and orientation with a total of 30 points. The higher the scores, the better their cognitive function. The cutoff value for distinguishing normal cognitive function from cognitive impairment was <26 points. To avoid the influence of years of education, 2 points were added to the total score of the scale for those whose years of education ≤6 years, 1 point was added to the total score of the scale for with years of education greater than 6 years and less than 12 years, and no extra points were added to the total score of the scale with years of education greater than 12 years or with total score greater than 30 points if the points were added. In this study, the Beijing edition of MoCA scale was adopted, and the retest reliability was 0.857.

##### 2.2.1.3 Muscle strength test

Muscle strength was assessed by performing grip strength and 30-s sit-to-stand test. Grip strength could reflect the overall strength and body function of the human body, and has high practicality and sensitivity. Participants needed to maintain an upright posture, with their arms stretched and dropping naturally to their side. The participants held the hand dynamometer and held it for 3–5 s with force. The left hand and right hand were tested for 3 times respectively, with an interval of 30 s for the test, and the average value was taken as their grip strength value. 30-s sit-to-stand had good reliability and validity for evaluating lower limb muscle strength in the elderly. A simple evaluation model of lower limb muscle strength in the elderly with 30-s sit-to-stand as the reference value has been initially established. Before the test, the participants stood in front of a chair about 43 cm high, with their hands crossed in front of their chest. Starting from the standing posture, the participants repeatedly stood up and sat down at the fastest speed after the timing began, and recorded the number of standing up for 30 s. During the test, the participants back needed to be straight and cannot touch the back of the chair. Their knee joint needed to be completely straight when they stood up.

#### 2.2.2 Information processing speed test

Part A of Trail Making Test (TMT-A) and Symbol Digit Modalities Test (SDMT) were conducted to evaluate information processing speed in this study. During the TMT-A, participants were required to connect the Arabic numerals 1–25 in sequence as quickly and correctly as possible. The shorter time and the fewer errors indicated that the participants had the faster information processing speed. The revised version of Professor Guo Qihao was used in this study, and the retest reliability was 0.890. During the SDMT, based on the existing number symbol template, the participants needed to fill in the corresponding symbol according to the given number within 90 s. The indicator of test analysis was 1 point for each correct symbol and 0.5 point for the inverted symbol. A lower score indicated a slower information processing speed. The retest reliability was 0.916.

##### 2.2.2.1 EEG signal collection

The EEG signal was collected by NCERP-190012, an EEG instrument equipped with preamplifier produced by Shanghai NCC electrical Co. Ltd., by using 16 unipolar leads, in which the sampling frequency was set at 500 Hz, high-pass filter was set at 0.3 Hz, low-pass filter was set at 30 Hz, and notch was set at 50 Hz. Based on the frequency, this instrument divided EEG into *δ*-frequency band (1-4 Hz), *θ*-frequency band (4-8 Hz), α1-frequency band (8–10.5 Hz), α2-frequency band (10.5-13 Hz), β1-frequency band (13-20 Hz), and β2-frequency band (20-30 Hz). The division of the frequency band adopted left closed and right open interval, and the critical value belonged to the high frequency band. The testing environment was the ventilated dark room with sound insulation design. Electronic devices that stimulated the senses, such as mobile phones, were not allowed in the room. After arriving at the dark room, the participants needed to familiarize themselves with the environment and adjust themselves to a comfortable sitting posture. Then, testers helped participants wear EEG caps and connect the electroencephalograph, and they also needed to adjust the impedance below 5kΩ. Following the 10/20 system electrode placement method prescribed by the Organization for Human Brain Mapping, the testers also set Fp1, Fp2, F3, F4, F7, F8, C3, C4, P3, P4, O1, O2, T3, T4, T5, and T6 leads, with GND as the earth electrode, and A1 as well as A2 that were in the position of both ear lobes as the reference electrode. At the same time, participants were told to keep awake, breathe steadily, keep their bodies as relaxing and still as possible, keep their hands at their sides, close their eyes, and avoid grinding their teeth or swallowing. After the waveform stabilized, the recording of 5 min EEG began.

### 2.3 Mathematical statistics

The toolbox was employed to eliminate artifact interference in the data segments, such as prominent eye movements. The electrode signals were categorized into the corresponding brain regions, including the orbitofrontal cortex (Fp1, Fp2), prefrontal cortex (F3, F4), lateral frontal lobe (F7, F8), central region (C3, C4), parietal region (P3, P4), occipital region (O1, O2), temporal region (T3, T4), and posterior temporal region (T5, T6). The EEG power indicator for each brain region was calculated as the sum of P left and P right.

The distribution of the data was assessed using frequency histograms. Continuous data that followed or approximated a normal distribution were described using the mean ± standard deviation, and group comparisons were conducted using independent sample *t*-tests. Categorical data were expressed as n (%) and analyzed for group differences using χ2 tests. Pearson correlation analysis was employed to examine the relationships between muscle strength, cognitive function, information processing speed, and EEG indicators. The Bonferroni correction was applied by dividing the significance level (0.05 or 0.01) by the number of comparisons (k) to adjust the significance threshold, thereby ensuring that the overall Type I error rate across multiple comparisons did not exceed 0.05 or 0.01. Additionally, several potential covariates were controlled for in the analysis, including age, gender, education level (illiterate, primary school, junior high, high school or higher), occupation (farmer, non-farmer), marital status (married/unmarried, divorced or widowed), alcohol consumption (yes/no), and body mass index (BMI, kg/m^2^).

A structural equation model was constructed using Amos Graphics to examine the relationships between muscle strength, information processing speed, EEG-specific indicators, and cognitive function (all variables were standardized prior to modeling). The model fit indices included RMR (Root Mean Square Residual), RMSEA (Root Mean Square Error of Approximation), GFI (Goodness of Fit Index), NFI (Normed Fit Index), and CFI (Comparative Fit Index). Parameter estimates for path analysis were derived using the non-parametric percentile bootstrap method (which makes no strict assumptions regarding variable distribution), with 5,000 bootstrap samples. The mediating effect was considered statistically significant if the 95% bias-corrected confidence interval (95% CI) for the product of the mediation paths did not include zero. Data analyses were conducted using SPSS Statistics 24.0 and Amos 24.0, with results reported to three decimal places (percentages rounded to two decimal places). All statistical inferences for parameters were conducted using two-tailed tests, with the significance level set at *α* = 0.05. *p*-values of <0.05, <0.01, and < 0.001 were considered statistically significant.

## 3 Results

### 3.1 Demographic differences of the elderly with different cognitive impairments

A total of 140 participants were included, with an average age of 72.51 ± 7.76 years and a BMI of 24.05 ± 2.93 kg/m^2^. Among them, 57.15% were male and 42.85% were female. The demographic characteristics of elderly individuals with mild cognitive impairment and those with moderate to severe cognitive impairment were compared, as shown in Table 1. Significant differences were found between the two groups in terms of age, marital status, education level, and occupation (*p* < 0.05 for all), while no significant differences were observed for other variables (*p* > 0.05 for all).

**Table 1:** Basic information table.

Variables	Total (*n* = 140)	Mild cognitive impairment (*n* = 73)	Moderate and severe cognitive impairment (*n* = 62)	Difference test
Age (years)	72.51 ± 7.76	70.18 ± 6.62	75.04 ± 8.14	*t* = −3.893, *P* < 0.001
BMI (kg/m^2^)	24.05 ± 2.93	24.46 ± 2.76	23.59 ± 3.06	*t* = 1.765, *p* = 0.08
Gender (%)				χ^2^ = 1.262, *p* = 0.306
Male	80 (57.15)	45 (61.64)	35 (52.24)	
Female	60 (42.85)	28 (38.36)	32 (47.76)	
Marital status (%)				χ^2^ = 7.793, *p* = 0.006
Married	112 (80)	65 (89.04)	47 (70.15)	
Unmarried, divorced or widowed	28 (20)	8 (10.95)	20 (29.85)	
Education level (%)				χ^2^ = 18.582, *P* < 0.001
Illiteracy	33 (23.57)	8 (10.95)	25 (37.31)	
Primary school	70 (50)	37 (50.68)	33 (49.25)	
Junior high school	30 (21.42)	23 (31.50)	7 (10.44)	
Senior middle school and over	7 (5)	5 (6.84)	2 (2.98)	
Occupation (%)				χ^2^ = 11.566, *P* < 0.001
Farmer	93 (66.42)	39 (53.42)	54 (80.59)	
Non-farmer	47 (33.57)	34 (46.57)	13 (19.40)	
Place of residence (%)				χ^2^ = 2.106, *p* = 0.183
Countryside	111 (79.28)	57 (78.08)	54 (80.59)	
City	29 (20.71)	16 (21.91)	13 (19.40)	
Smoking (%)				χ^2^ = 3.028, *p* = 0.111
Yes	50 (35.71)	31 (42.46)	19 (28.35)	
No	90 (64.28)	42 (57.53)	48 (71.64)	
Drinking (%)				χ^2^ = 4.2478, *p* = 0.053
Yes	52 (37.14)	33 (45.20)	19 (28.35)	
No	88 (62.85)	40 (54.79)	48 (71.64)	
Whether there existed the decline in hearing (%)				χ^2^ = 1.982, *p* = 0.371
Yes	68 (48.60)	26 (41.93)	17 (54.83)	
No	72 (51.40)	36 (58.13)	14 (45.16)	
Whether there was a decline in vision (%)				χ^2^ = 0.1341, *p* = 0.725
Yes	92 (65.71)	49 (67.12)	43 (64.17)	
No	48 (34.28)	24 (32.87)	24 (35.82)	
History of chronic disease (%)				χ^2^ = 0.001, *p* = 0.999
Yes	98 (70)	22 (30.13)	20 (29.85)	
No	42 (30)	51 (69.86)	47 (70.14)	
Social activities				χ^2^ = 1.982, *p* = 0.316
≥2 types	69 (49.29)	39 (53.42)	30 (44.78)	
<2 types	71 (50.71)	34 (46.58)	37 (55.22)	

### 3.2 The difference of information processing speed in the elderly with different degrees of cognitive impairment

The elderly with cognitive impairment in this study were divided into mild cognitive impairment and moderate and severe cognitive impairment according to MoCA scores. It was found that there existed significantly statistical differences in SDMT scores and the time consumption of TMT-A of the elderly with cognitive impairment among different levels of MoCA score (all *p* < 0.001) (Table 2).

**Table 2:** Comparison of information processing speed in the elderly with cognitive impairment.

Variables	Total (*n* = 140)	Mild cognitive impairment (*n* = 73)	Moderate and severe cognitive impairment (*n* = 62)	Difference test
SDMT score	18.07 ± 8.05	22.46 ± 7.32	13.28 ± 5.78	*t* = 8.182, *P* < 0.001^***^
Time consumption of TMT-A	113.81 ± 62.51	93.34 ± 49.64	136.10 ± 67.63	*t* = −4.289, *P* < 0.001^***^

*, **, and *** Meant *P* < 0.05, *P* < 0.01, and *P* < 0.001, respectively.

### 3.3 Relationship between MoCA score and information processing speed in the elderly with cognitive impairment

**Figure 3:**
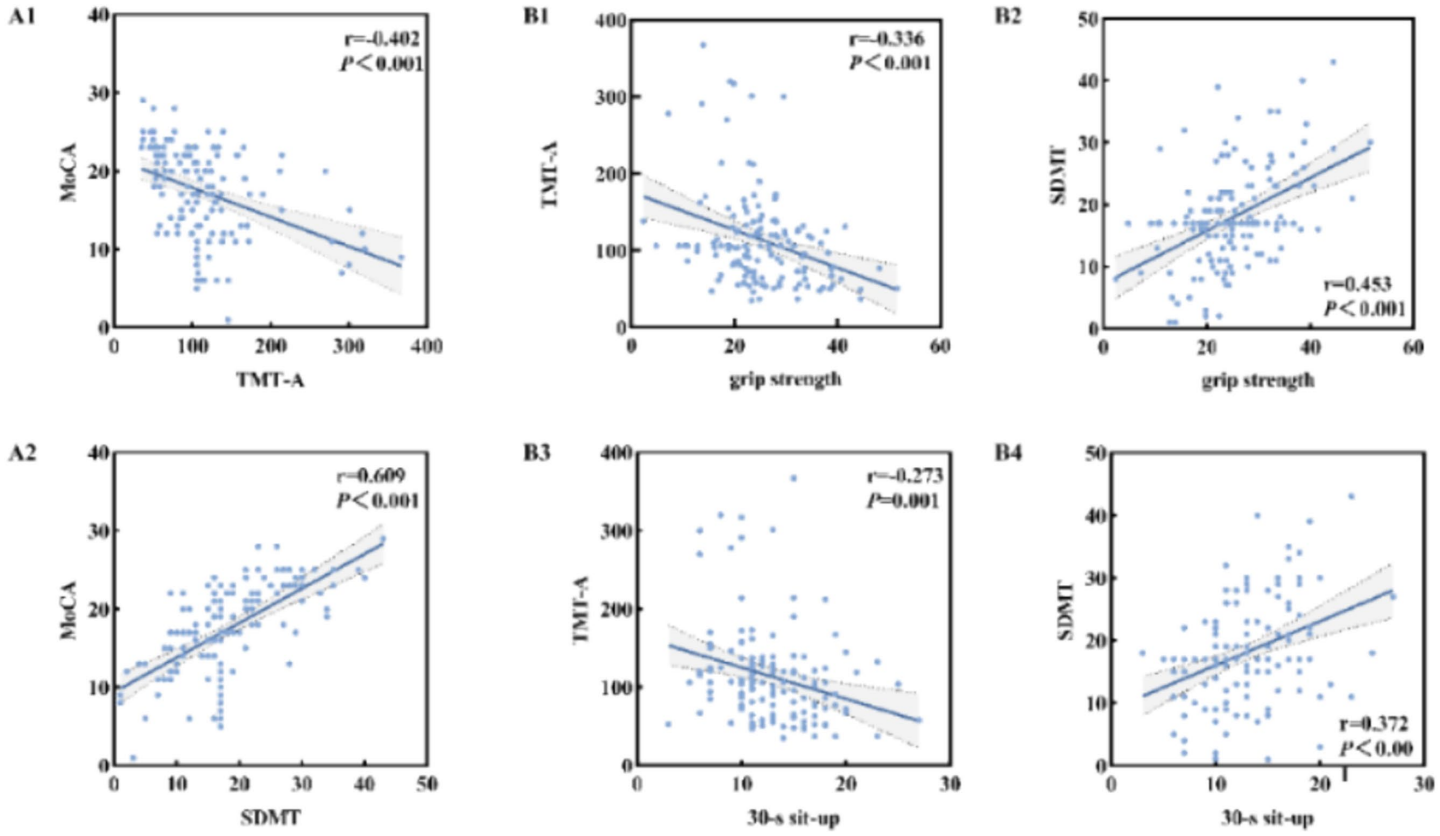
Relationship between information processing speed and MoCA score in the elderly with cognitive impairment **(A1,A2)**; Relationship between muscle strength and information processing speed in the elderly with cognitive impairment **(B1–B4)**.

After controlling confounding factors, Pearson correlation analysis was used to investigate the relationship between MoCA score and information processing speed. As shown in Figure 3, time consumption of TMT-A was significantly negatively correlated with MoCA score (*r* = −0.402, *p* < 0.01), while SDMT score was significantly positively correlated with MoCA score (*r* = 0.609, *p* < 0.01).

### 3.4 Relationship between muscle strength and information processing speed in the elderly with cognitive impairment

After controlling confounding factors, Pearson correlation analysis was used to investigate the relationship between muscle strength and information processing speed. As shown in Figure 3, the time consumption of TMT-A was significantly negatively correlated with grip strength (*r* = −0.336, *p* < 0.01) and 30-s sit-to-stand (*r* = −0.273, *p* < 0.01). SDMT score was significantly positively correlated with grip strength (*r* = −0.336, *p* < 0.01) and 30-s sit-to-stand (*r* = 0.372, *p* < 0.01).

### 3.5 Correlation between information processing speed, MoCA score and the EEG indicators in whole brain area

Table 3 showed the correlation between the EEG indicators in whole brain area, MoCA score, time consumption of TMT-A, and SDMT score. The results showed that the power value of α1 in whole brain area was significantly correlated with MoCA score, time consumption of TMT-A and SDMT score.

**Table 3:** Correlation coefficients between the EEG indicators of each brain region and SDMT score, MoCA score, and time consumption of TMT-A.

Power value indicators	MoCA score	Time consumption of TMT-A	SDMT score
δ(FP1 + FP2)	−0.090	0.077	0.002
θ(FP1 + FP2)	−0.281^** a^	0.105	−0.195^*^
α1(FP1 + FP2)	0.222^** a^	−0.206^*^	0.227^** a^
α2(FP1 + FP2)	0.162	−0.202^*^	0.160
β1(FP1 + FP2)	0.037	−0.078	0.112
β2(FP1 + FP2)	−0.147	0.069	−0.057
δ(F3 + F4)	−0.132	0.073	0.046
θ(F3 + F4)	−0.213^*^	0.001	−0.080
α1(F3 + F4)	0.241^** a^	−0.208^*^	0.239^** a^
α2(F3 + F4)	0.158	−0.218^** a^	0.168^*^
β1(F3 + F4)	0.031	−0.096	0.128
β2(F3 + F4)	−0.112	0.050	−0.006
δ(C3 + C4)	−0.193^*^	0.108	0.010
θ(C3 + C4)	−0.119	−0.037	−0.113
α1(C3 + C4)	0.293^** a^	−0.225^** a^	0.217^*^
α2(C3 + C4)	0.242^** a^	−0.229^** a^	0.159
β1(C3 + C4)	0.151	−0.157	0.117
β2(C3 + C4)	0.018	−0.033	−0.007
δ(P3 + P4)	−0.189^*^	0.083	0.022
θ(P3 + P4)	−0.059	−0.067	−0.088
α1(P3 + P4)	0.298^** a^	−0.219^** a^	0.197^*^
α2(P3 + P4)	0.244^** a^	−0.210^*^	0.144
β1(P3 + P4)	0.188^*^	−0.160	0.097
β2(P3 + P4)	0.095	−0.065	0.005
δ(O1 + O2)	−0.123	0.123	0.072
θ(O1 + O2)	−0.142	−0.031	−0.090
α1(O1 + O2)	0.235^** a^	−0.187^*^	0.192^*^
α2(O1 + O2)	0.208^*^	−0.192^*^	0.161
β1(O1 + O2)	0.131	−0.139	0.092
β2(O1 + O2)	−0.009	−0.038	−0.031
δ(F7 + F8)	−0.109	0.036	0.044
θ(F7 + F8)	−0.035	−0.089	−0.068
α1(F7 + F8)	0.312^** a^	−0.233^** a^	0.213^*^
α2(F7 + F8)	0.272^** a^	−0.220^** a^	0.164
β1(F7 + F8)	0.199^*^	−0.174^*^	0.119
β2(F7 + F8)	0.094	−0.072	0.017
δ(T3 + T4)	−0.203^*^	0.112	−0.022
θ(T3 + T4)	−0.219^**^	0.127	−0.074
α1(T3 + T4)	0.197^*^	−0.213^*^	0.233^** a^
α2(T3 + T4)	0.141	−0.136	0.226^** a^
β1(T3 + T4)	−0.038	0.040	0.143
β2(T3 + T4)	−0.163	0.130	−0.010
δ(T5 + T6)	−0.155	0.127	−0.001
θ(T5 + T6)	−0.124	−0.066	−0.102
α1(T5 + T6)	0.273^** a^	−0.221^** a^	0.204^*^
α2(T5 + T6)	0.241^** a^	−0.216^*^	0.164
β1(T5 + T6)	0.134	−0.155	0.088
β2(T5 + T6)	−0.039	−0.043	−0.047

*, **, and *** meant *P* < 0.05, *P* < 0.01, and *P* < 0.001, respectively; ^a^ Significance results after Bonferroni correction.

### 3.6 Potential relationship pathway of muscle strength, information processing speed and EEG specific indicators

**Figure 4:**
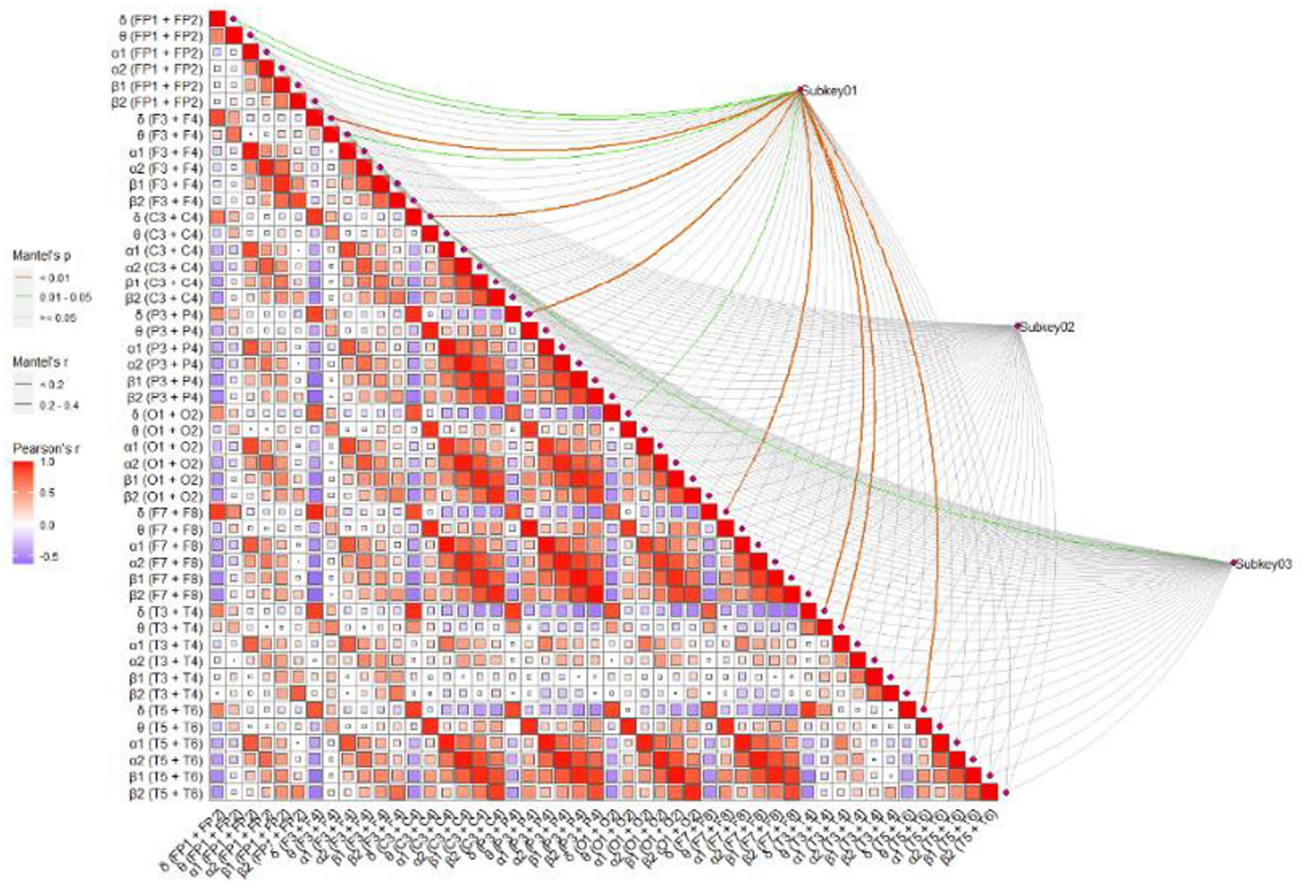
The correlation between EEG indicators and MoCA, TMT-A, and SDMT. Subkey01, MoCA; Subkey02, TMT-A; Subkey03, SDMT.

**Figure 5:**
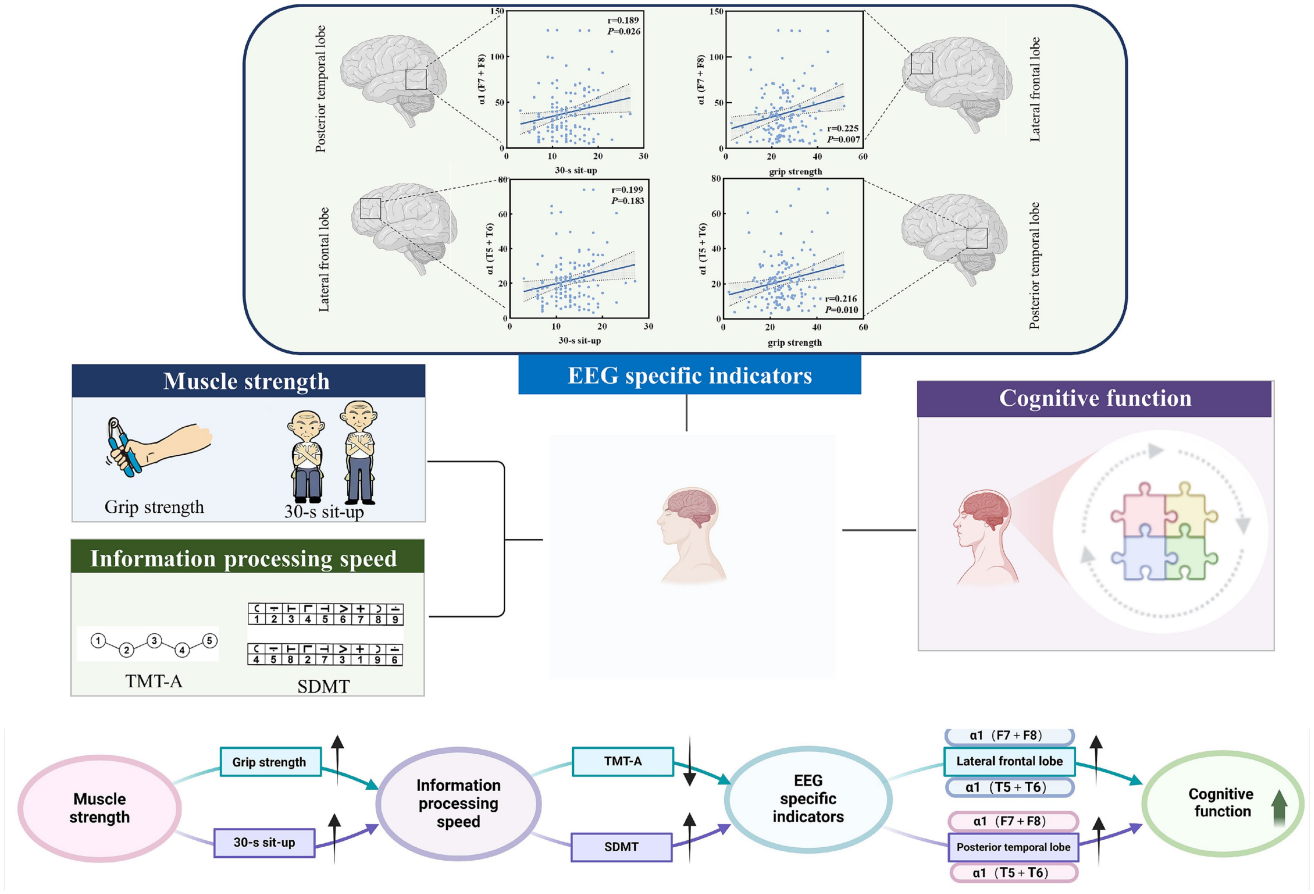
Potential pathways for muscle strength, information processing speed, and EEG-specific indicators.

To further screen the EEG specific indicators that were sensitive to muscle strength, Pearson correlation coefficient was used to investigate the relationship between muscle strength and EEG specific indicators. As shown in Figure 4, the power value of α1 (F7 + F8) and α1 (T5 + T6) were both the sensitive EEG indicators for the muscle strength and information processing speed (Table 4 and Figure 5).

**Table 4:** Correlation between EEG power value indicators and muscle strength as well as information processing speed.

Power value indicators	Muscle strength		Information processing speed		Cognitive abilities
	Grip strength	30-s sit-to-stand	Time consumption of TMT-A	SDMT score	MoCA score
α1(FP1 + FP2)	0.184^*^	0.111	−0.206^*^	0.227^** a^	0.222^** a^
α1(F3 + F4)	0.184^*^	0.151	−0.208^*^	0.239^**^	0.241^** a^
α1(C3 + C4)	0.213^*^	0.146	−0.225^** a^	0.217^*^	0.293^** a^
α1(P3 + P4)	0.217^** a^	0.151	−0.219^** a^	0.197^*^	0.298^** a^
α1(O1 + O2)	0.205^*^	0.165	−0.187^*^	0.192^*^	0.235^** a^
α1(F7 + F8)	0.225^** a^	0.188^*^	−0.233^** a^	0.213^*^	0.312^** a^
α1(T3 + T4)	0.136	0.166	−0.213^*^	0.233^** a^	0.197^*^
α1(T5 + T6)	0.216^*^	0.199^*^	−0.221^** a^	0.204^*^	0.273^** a^
α2(C3 + C4)	0.255^** a^	0.142	−0.229^** a^	0.159	0.242^** a^
α2(P3 + P4)	0.260^** a^	0.145	−0.210^*^	0.144	0.244^** a^
α2(O1 + O2)	0.242^** a^	0.137	−0.192^*^	0.161	0.208^*^
α2(F7 + F8)	0.266^** a^	0.193^*^	−0.220^** a^	0.164	0.272^** a^
α2(T5 + T6)	0.248^** a^	0.183^*^	−0.216^*^	0.164	0.241^** a^
β1(F7 + F8)	0.186^*^	0.165	−0.174^*^	0.119	0.199^*^
θ(FP1 + FP2)	−0.304^** a^	−0.164	0.105	−0.195^*^	−0.281^** a^

*, **, and *** meant *P* < 0.05, *P* < 0.01, and *P* < 0.001, respectively; ^a^ Significance results after Bonferroni correction.

### 3.7 Pathway construction of muscle strength, information processing speed, EEG-specific indicators, and cognitive function

**Figure 6:**
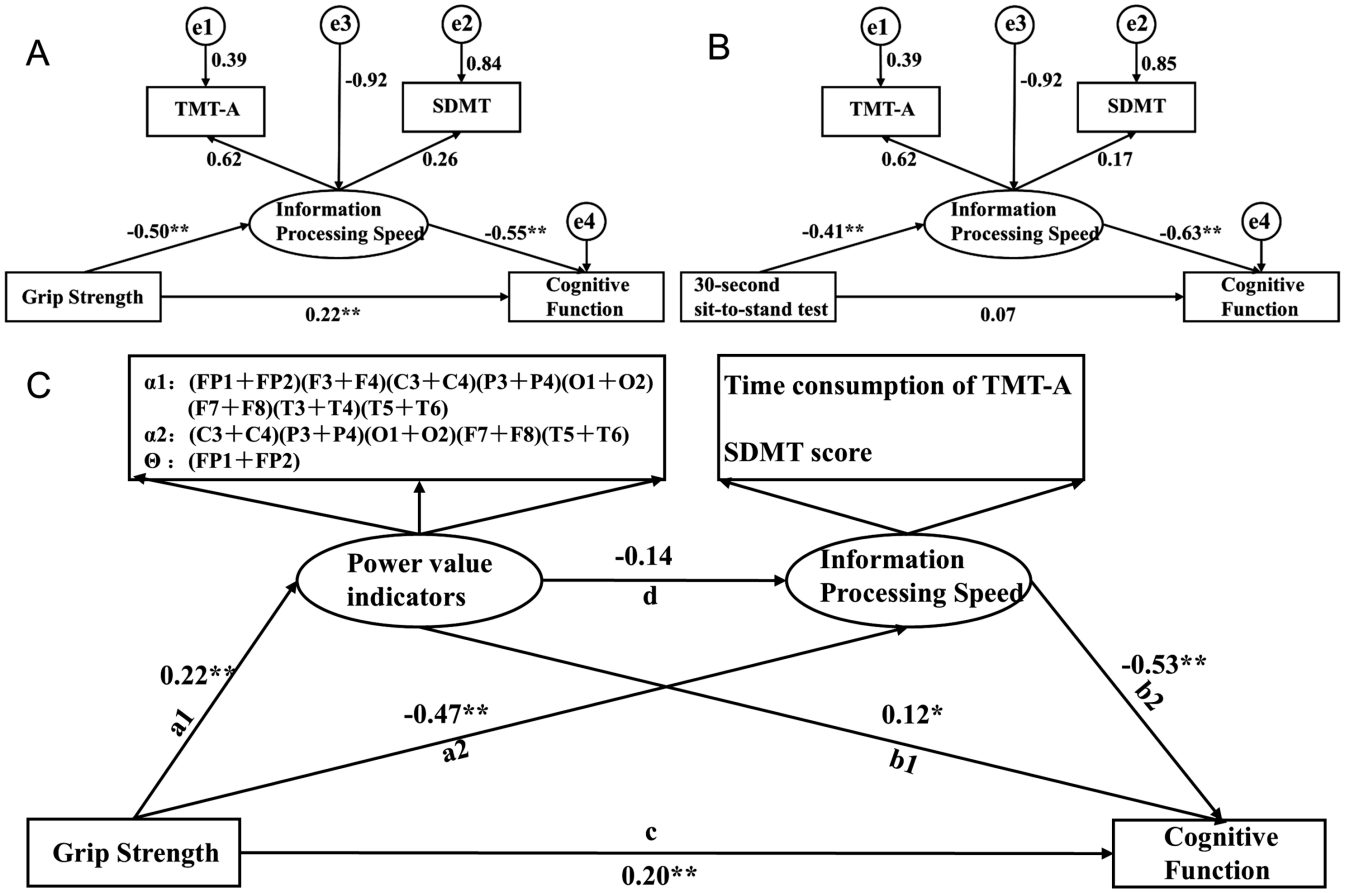
Pathway construction of muscle strength, information processing speed, EEG-specific indicators, and cognitive function; **(A)** Mediation Model 1 of grip strength, information processing speed, and cognitive function; **(B)** Mediation Model 2 of 30-second sit-to-stand, information processing speed, and cognitive function; **(C)** Structural equation Model 3 with EEG-specific indicators and information processing speed as serial mediators.

The path analysis is shown in Figure 6, with the results of the mediating effect test presented in Table 5. First, we constructed a mediation model involving muscle strength, information processing speed, and cognitive function. The results of Model 1 show that grip strength has a direct effect of 45.13% on MOCA scores (*B* = 0.137, 95% CI: 0.011, 0.267), indicating that higher grip strength is associated with higher MOCA scores, and this direct effect is significant. Information processing speed, as a latent variable, mediates 46.87% of the effect (*B* = 0.187, 95% CI: 0.108, 0.318), with all path coefficients being significant, and the path coefficient for SDMT scores being higher. Model 1 is a partial mediation model. The results of Model 2 show that the direct effect of the 30-s sit-to-stand test on MOCA scores is not significant (*B* = 0.093, 95% CI: −0.117, 0.238). Information processing speed, as a latent variable, mediates 79.10% of the effect (*B* = 0.352, 95% CI: 0.185, 0.625), with all path coefficients being significant, and the path coefficient for SDMT scores being higher. Model 2 is a full mediation model. The results indicate that information processing speed mediates the relationship between muscle strength and MOCA scores, with a higher impact from SDMT scores.

**Table 5:** Bootstrap test results of mediating effects.

Model	Type of effect	*B*	95%CI	*P*	SE	Proportion of effect (%)
Model 1	Total effect	0.339	[0.242,0.424]	<0.001	0.047	100
	Direct effect	0.153	[0.011,0.267]	0.035	0.058	45.13
	Mediating effect	0.187	[0.108,0.318]	<0.001	0.051	46.87
Model 2	Total effect	0.445	[0.213,0.679]	0.001	0.119	100
	Direct effect	0.093	[−0.117,0.238]	0.403	0.111	20.89
	Mediating effect	0.352	[0.185,0.625]	<0.001	09.110	79.10
Model 3	Total effect	0.339	[0.242,0.424]	<0.001	0.047	100
	Direct effect	0.137	[0.031,0.382]	0.014	0.058	40.41
	Path a1-b1	0.019	[0.003,0.047]	0.018	0.011	5.60
	Path a2-b2	0.170	[0.096,0.292]	<0.001	0.052	50.00
	Path a1-d-b2	0.013	[−0.001,0.042]	0.079	0.012	3.99

Based on the findings from Section 2.6 and the results of Models 1 and 2, there is a close relationship between muscle strength, information processing speed, and cognitive function, with a stronger relationship between grip strength and EEG-specific indicators. Therefore, based on the interrelationships among muscle strength, EEG-specific indicators, information processing speed, and cognitive function in elderly individuals with cognitive impairments, we further constructed Structural Equation Model 3, with grip strength as the independent variable, cognitive function scores as the dependent variable, and EEG-specific indicators and information processing speed as serial mediators. The model fit indices showed CMIN/df = 12.98, RMR = 45.896, RMSEA = 0.293, GFI = 0.572, NFI = 0.742, and CFI = 0.756. Grip strength has a direct effect of 40.41% on MOCA scores (*B* = 0.137, 95% CI: 0.031, 0.382), indicating that higher grip strength is associated with higher MOCA scores, and this direct effect is significant. The path a1-b1 results show that EEG-specific indicators, as a latent variable, mediate 5.60% of the effect (*B* = 0.019, 95% CI: 0.003, 0.047), with all path coefficients being significant, and the path coefficient for the α1 frequency band being higher. The path a2-b2 results show that information processing speed, as a latent variable, mediates 50.00% of the effect (*B* = 0.137, 95% CI: 0.096, 0.292), with all path coefficients being significant, and the path coefficient for SDMT scores being higher. The path a1-d-b2 results show that the joint mediating effect of EEG-specific indicators and information processing speed is not significant (*B* = 0.013, 95% CI: −0.001, 0.042). Specifically, the effect between EEG-specific indicators and information processing speed is not significant (*B* = −0.702, 95% CI: −1.811, 0.193). The results indicate that EEG-specific indicators and information processing speed can mediate the relationship between grip strength and MOCA scores, with partial mediation, and the impact of information processing speed being stronger. However, the joint mediating effect of EEG-specific indicators and information processing speed approaches significance, which may be due to the interaction between EEG-specific indicators.

## 4 Discussion

The findings of this study suggest that muscle strength, particularly grip strength, may serve as a significant predictor of information processing speed in elderly individuals with cognitive impairments. These results substantiate our hypothesis and offer novel evidence regarding the role of EEG-specific indicators in individuals with cognitive impairment. Prior research has shown that muscle strength is a reliable predictor of cognitive decline in individuals with cognitive impairment (Sui et al., 2020; Filardi et al., 2022). Furthermore, other cross-sectional studies have indicated that both grip strength and lower limb muscle strength are predictive of cognitive impairment (Filardi et al., 2022; Storoschuk et al., 2023).

The potential relationship between grip strength and information processing speed may be attributed to the regulation of muscle function by the nervous system. Some studies have suggested that the decline in grip strength is primarily attributable to age-related changes in the muscular system, while others propose that it is due to a deterioration in the function of the nervous and motor systems in the elderly, including impaired neuromuscular activation and reduced motor unit recruitment (Carson, 2018; Shinohara et al., 2003). This phenomenon, in which impairment of the nervous and motor systems contributes to the decline in grip strength during the aging process, may also be linked to early and progressive cognitive impairment (Bishop et al., 2010). These findings are consistent with the results of the present study. Our results indicate that greater muscle strength is associated with enhanced activation of *α*1 (F7 + F8), α1 (T5 + T6), and faster information processing. Increased muscle strength was associated with greater activation of α1 (F7 + F8) and α1 (T5 + T6), reduced time consumption on TMT-A, and higher SDMT scores. A cross-sectional study involving over 40,000 participants from the UK Biobank explored the association between high grip strength and increased gray matter volume, particularly in the subcortical regions and temporal cortex. Furthermore, the study confirmed that the correlation between processing speed and grip strength was the strongest (Jiang et al., 2022).

The present study found that information processing speed was positively correlated with increased activation of the α1-frequency band in the lateral frontal lobes and posterior temporal lobes. The α-frequency band is one of the most significant EEG rhythms in the awake state and may underpin top-down cognitive processes (Berger, 1935). Previous studies have shown that the thalamus serves as the primary pacemaker for the α-frequency band. The classic posterior α-frequency band is driven by the pulvinar and lateral geniculate nucleus (LGN) (Saalmann et al., 2012; Lorincz et al., 2009; Vijayan and Kopell, 2012). Within the cortex, it is widely accepted that the α-frequency band originates from the subgranular zone, driven by pyramidal cells in layer V (Van Kerkoerle et al., 2014; Buffalo et al., 2011). The power of the α-frequency band and its correlation with task reaction time have been linked to impaired attention in the elderly, with these features serving as potential neurobiomarkers to differentiate between patients with MCI and cognitively healthy individuals (Chen et al., 2020). A decrease in the α-frequency band in patients with AD and MCI is thought to reflect cortical inhibition and is inversely related to hemodynamic measures of neurological activity (Jafari et al., 2020). The α1-frequency band is widely distributed across brain networks and is thought to play a role in brain arousal and spatial attention (Foster et al., 2017; Klimesch, 2012). The α2-frequency band may reflect an inhibitory control mechanism that regulates the intake of visual information (Worden et al., 2000). The reduced frequency of the α1-frequency band in the resting state in MCI may indicate diminished cortical inhibition, leading to heightened sensitivity to environmental novelty, or greater global brain arousal, which could serve as a compensatory mechanism in these disease states. In this study, α1 (F7 + F8) and α1 (T5 + T6) were negatively correlated with TMT-A completion time in patients with MCI and positively correlated with SDMT scores, which is consistent with previous studies showing that the α1-frequency band may be a valuable marker for monitoring cognitive decline in MCI patients. Previous studies have reported that attenuation of the α1-frequency band in resting states is associated with cognitive decline in Alzheimer’s disease (Damoiseaux et al., 2008). The α1-frequency band of EEG has also been found to predict cognitive performance in healthy adults (Maclean et al., 2012) and is thought to exert an inhibitory function on cortical activity, likely through selective activation of brain networks when needed (Klimesch, 2012). Therefore, the correlation between attenuation of the α1-frequency band and cognitive decline in MCI patients may suggest that disruption of its inhibitory function leads to nonspecific cortical stimulation, thereby impairing attention and information processing speed.

Through the investigation of the aforementioned relationships, this study identified a potential pathway linking muscle strength to cognitive function via EEG-specific indicators and information processing speed, thereby contributing valuable insights to advancing the field of cognitive research in the elderly.

## 5 Limitations and future directions

First, the study sample consisted of elderly individuals residing in nursing homes. Due to their advanced age, it is likely that their brains have experienced varying degrees of atrophy, which may limit the generalizability of our findings. Future research should aim to validate these findings in a younger cohort of elderly individuals living independently in the community. Second, while our sample size meets the required standards, it remains relatively small. Despite further residual adjustments to improve model fit, the structural equation model revealed that some fit indices were suboptimal, likely due to the complex interrelationships among the EEG indicators. Third, factors such as heart disease, diabetes, hypertension, and education level may influence the results, and should therefore be accounted for as potential confounding variables in future studies. Finally, it is worth noting that recent studies favor median frequency (MDF) in alpha band or dominant alpha frequency as a more precise marker for improvements in information processing speed. Future studies should consider incorporating MDF for a more comprehensive analysis.

## 6 Conclusion

Muscle strength, EEG-specific indicators, information processing speed, and cognitive function are closely interconnected in elderly individuals with cognitive impairment. The pathway linking muscle strength, EEG-specific indicators, information processing speed, and cognitive function may provide valuable insights for advancing research on cognitive health in the elderly. To identify and mitigate cognitive decline in the elderly, grip strength tests, the 30-s sit-to-stand test, the Trail Making Test, the Symbol Digit Modalities Test, and EEG assessments should be integrated into routine screenings in nursing homes and communities.

## Data Availability

The raw data supporting the conclusions of this article will be made available by the authors, without undue reservation.
